# Perceptions of dietary sugar consumption among public housing residents using a modified qualitative photovoice methodology

**DOI:** 10.1186/s12889-025-22391-2

**Published:** 2025-04-08

**Authors:** Mabeline Velez, Brenda Heaton, Chelsey Solar, Yinette Fuertes, Belinda Borrelli, Raul I. Garcia, Lisa M. Quintiliani

**Affiliations:** 1 Department of Health Policy & Health Services Research, Boston University Henry M. Goldman School of Dental Medicine, Boston, MA USA; 2 Department of Epidemiology, Boston University School of Public Health, Boston, MA USA; 3 VA Boston Healthcare System, Boston, MA USA; 4 Section of General Internal Medicine, Department of Medicine, Boston University, Boston Medical Center, Boston, MA USA; 5 Department of Community Health Sciences, Boston University School of Public Health, Boston, MA USA; 6 School of Dentistry, University of Utah, 530 Wakara Way, Salt Lake City, UT USA

**Keywords:** Dietary sugars, Public housing, Community-based participatory research, Qualitative research, Photovoice

## Abstract

**Background:**

Consumption of dietary sugar (e.g. sugar-sweetened beverages and high sugar foods) is a predominant contributor to chronic health conditions, particularly in communities of low socio-economic position. Our objective was to explore social contextual influences on dietary sugar consumption among public housing residents in Boston, MA.

**Methods:**

This study employed the use of photovoice, a qualitative technique involving participant photography and narratives. Due to the COVID-19 pandemic, we conducted photovoice methods using Zoom. Adult residents of two public housing developments were invited to participate in pairs of online group sessions. The first session provided training on photovoice methodology and a discussion of example photographs and written narratives. Over the ensuing two weeks, participants took or identified stock photos as visual examples of personally-experienced barriers and facilitators of avoiding sugary foods and beverages. During the second session, study staff facilitated development of verbal narratives via group discussion. A total of 18 sessions were audio recorded, transcribed, and double-coded for themes.

**Results:**

Participants (*n* = 49) were predominantly women and identified as either Hispanic (61.2%) or non-Hispanic Black (30.6%). Approximately half of participants (51.1%) reported consuming sugar-sweetened beverages at least once per day. Qualitative analysis revealed participant-identified influences on dietary sugar consumption across multiple domains of influence, including individual preferences, beliefs, or circumstance, the social environment, the physical environment, and the macro environment.

**Conclusions:**

The multiple social contextual influences on dietary sugar consumption identified in this study, particularly centrality of the home, cultural influences, individual-level sabotaging factors, may be useful for development of culturally tailored health promotion messaging and intervention through multiple channels.

## Background

Consumption of sugar-sweetened beverages and foods is a major risk factor for developing chronic health conditions [1–3]. In the United States, 63% of adults consume sugary drinks at least daily, which includes regular soda, sweetened fruit drinks, sport/energy drinks, and sweetened coffee/tea drinks [1]. While sugar-sweetened beverage consumption is high, national data reveals that the majority of calories from added sugar are not from beverages, but from foods [4]. Thus, approaches to chronic disease prevention aimed at decreasing sugar intake need to incorporate both beverages and foods for maximum impact. Additionally, significant disparities in sugar consumption exist. Racial/ethnic minority populations and people with low incomes are more likely to consume sugar-sweetened beverages and foods more frequently and in greater amounts compared to those with higher incomes [2]. For this reason, any effort aimed at addressing the well-documented population disparities in chronic disease would need to address the population-specific forces that drive sugar consumption.

Public housing is a nationwide, governmental model for housing the urban poor. Public housing developments are characterized by demographic profiles reflective of lower socioeconomic position, including racial/ethnic minority status, and a majority of whom live well below the federal poverty line [5]. The poor health of residents in public housing developments is due in large part to higher-level systemic and structural factors, rather than any preexisting health conditions [6]. In addition to the social environment created by the residents themselves, housing developments are physically located in neighborhoods that have multiple objective (distance to and density of food stores) and perceived (perceptions of availability of healthier food) dimensions that can create barriers to healthy eating behaviors.

Generally, communities at high-risk of poor health outcomes are often those communities that face unique challenges to maintaining good health, such as objective and perceived restricted access to healthy foods [7], reduced opportunities for physical activity [8], and social environments that reinforce poor health behaviors [9]. For this reason, community-engaged methods can be particularly useful in identifying population-specific barriers that may impact translation of evidence-based interventions [10]. Qualitative methods offer unique advantages that complement quantitative approaches, such as capturing cultural differences, and providing a deeper understanding of the social context influencing health behaviors. One such novel strategy is photovoice, a method aimed at engaging community participation in the identification and assessment of community needs [4–6]. Photovoice methods that help to elicit this information and context can help us in the development of a tailored health promotion intervention by allowing participants to express their perspectives through visual narratives. This method not only enhances participant engagement but also ensures that health promotion messages and interventions are culturally relevant and resonate with the target population. Photovoice methods afford researchers the opportunity to have access to information about people in their real-world contexts (e.g., homes, interactions with family members, neighborhood events) through enabling community members to take photographs of their environment and provide an accompanying narrative. In this way, the process directly engages community members, allowing them to more fully explain their perceptions through the visual cues a photograph provides. Prior research has employed photovoice methods to describe influences on eating behaviors in a variety of populations, including rural women [11], public housing residences [12], and urban communities in Canada [13]. However, prior research applying this method has not placed a primary focus on the consumption of sugar-sweetened beverages and foods—a major common risk factor for multiple chronic disease outcomes [6].

**Fig. 1:**
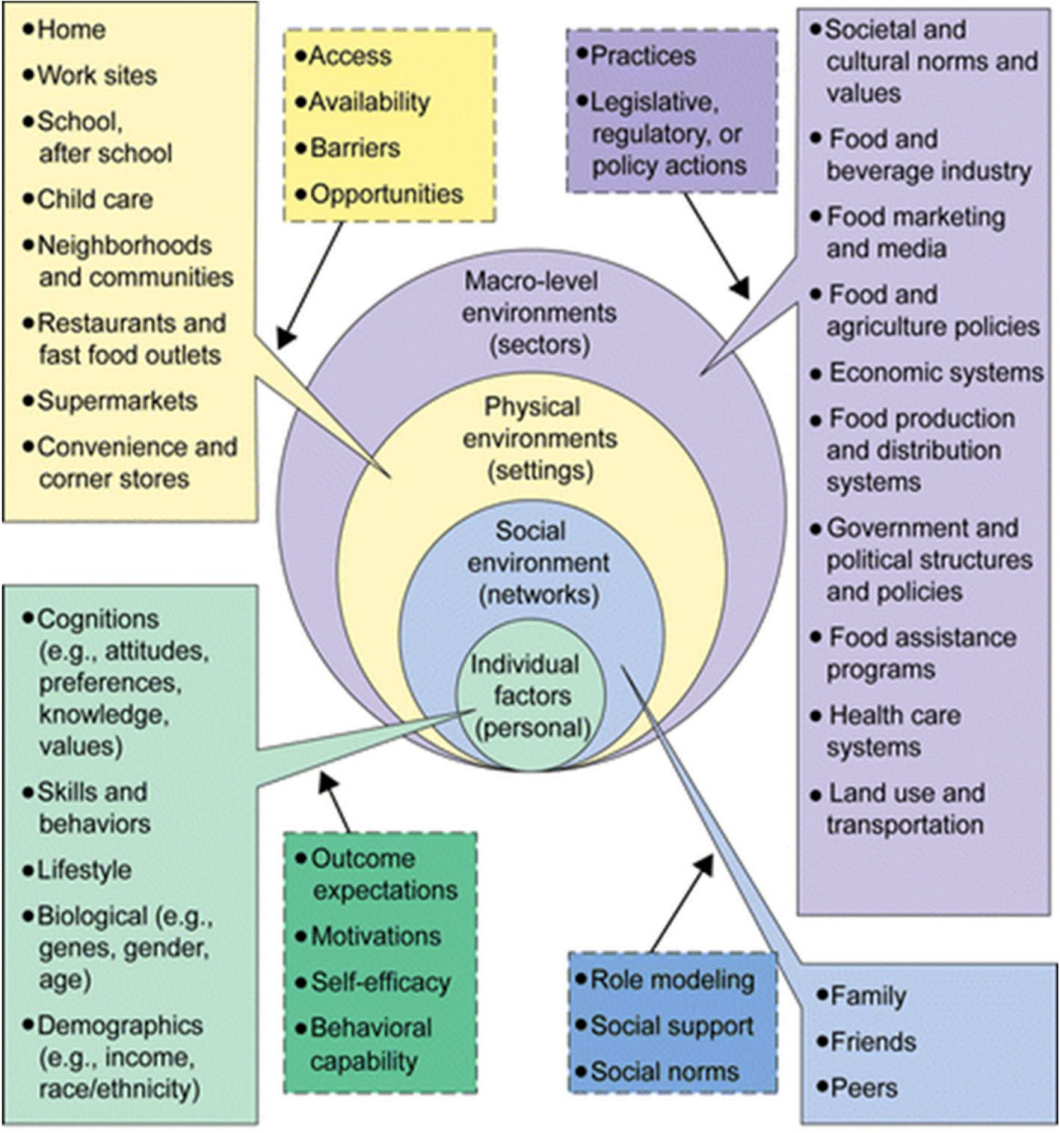
The Ecological Framework. This figure depicts the multiple levels of influence operating on individual food choices, known as the Ecological Framework. This figure is used with permission from the author, Dr. Mary Story

In this study, we focus on residents of public housing, a population suffering from a disproportionately higher burden of chronic disease with well-documented challenges in obtaining the necessary resources for maintaining good health [6]. The approach is guided by the perspective that multiple levels of influence are exerted on food and beverage consumption behaviors as depicted in the ecological framework: the individual level, social environment, physical environment and macro level (see Fig. 1) [14]. The objective of this qualitative study was to examine, through the use of photovoice, multi-level factors that influence sugary food and beverage consumption among a low-income, urban population of public housing residents, with the accompanying goal of being able to develop population-specific messaging around reduction of sugary food and beverage consumption.

## Methods

We recruited participants from among residents living in a family-designated public housing development located in Boston, Massachusetts. Study conduct occurred from February to November of 2020. Our intended sample size of 40 participants was guided by our desire to include both English- and Spanish-speaking adults, and to ensure that there would be some variability by age. Further, we had confidence that we could attain thematic saturation based on our prior experiences applying photovoice methods in similar populations [12]. We worked with our partner in the Boston Housing Authority and the development’s management team to identify feasible and effective strategies for participant recruitment. Three female bilingual study staff conducted door-to-door knocking and posted informational flyers throughout the development’s community spaces. Individuals were considered eligible if they were adult residents of the development with no intention of moving in the next three months, and spoke either English or Spanish. We provided verbal and written explanations of the study to interested residents; residents were then asked to provide verbal agreement to participate. The Boston University Medical Campus Institutional Review Board approved our study protocol (H-39298). Upon enrollment, study staff conducted a brief, in-person structured interview to assess eligibility criteria and to complete a questionnaire that assessed socio-demographics, sugary food and beverage consumption [15], sugar-sweetened beverage-related [15] knowledge self-efficacy to limit sugar-sweetened beverages and sugary foods, food insecurity [16], and self-reported measures of overall health and oral health.

### Photovoice methods

The photovoice method followed a multi-step process. First, participants were asked to join a training session during which participants were 1) introduced to photovoice using example photos from previous studies [8, 17] and applicable photos found online; 2) asked to verbally describe how these photographs related to their consumption of sugary foods and beverages; 3) instructed to take their own photographs with personal smartphones over a 2-week period following these prompts: “*What makes it hard to avoid sugary drinks and/or foods*? *What makes it easy to avoid sugary drinks and/or foods? Think about things in your life and in your community,*” and 4) provided with instructions on the proper technique for capturing photos, including the importance of respecting the privacy of people they may include in photographs. Once completed, participants were given two weeks to collect as many photos as they liked. Study staff members checked in with participants after one week by text or phone in order to remind them to collect photographs and answer any questions. At the end of the two weeks, we asked participants to submit their photographs via email or text message and to select one-to-two of their most meaningful photos to share during a discussion session. During the discussion session, a study staff member used a semi-structured moderator’s guide to allow participants to share and discuss their selected photos and to ask probing questions to explore how the photograph was related to their sugary food and beverage consumption (e.g., *What does this picture tell us about what makes it hard to avoid sugary drinks and/or foods? What does this picture tell us about what makes it easy to avoid sugary drinks and/or foods?)*. Both the training and discussion Sessions were each 60 min in length and audio recorded.

In response to the emerging COVID-19 pandemic at the time data collection occurred (Spring/Summer 2020), we tailored the approach to photovoice methods in a way that would facilitate participation, encourage pandemic safety, and completion of the study objectives. Specifically, all training and discussion sessions occurred via Boston University’s institutional Zoom account. Additionally, participants were instructed to take pictures outside and around the community if they felt safe doing so; otherwise participants could collect online photographs that they felt represented their ideas. In addition, study staff encouraged participants to develop verbal narratives to accompany the photographs, rather than written narratives, a traditional aspect of photovoice methodology. We did this by verbally summarizing the stated narrative and reflecting back to ensure that we summarized correctly, allowing for the participant to make additional clarifications. In the case that they clarified or revised the narrative, we summarized and reflected back again to arrive at a final narrative [18]. Immediately following the conclusion of the group, we discussed the content and any need to augment future discussions based on what seemed to be emerging from groups. Once all groups were completed and transcripts were generated, we embarked on a formal analysis and the development of codes/code book [17].

### Data analysis

The enrollment survey was analyzed primarily using descriptive statistics, including means, counts, and frequencies. Additionally, scores were calculated using published procedures for validated scales and questionnaires, e.g. sugar-sweetened beverage-related knowledge, and self-efficacy to limit sugar-sweetened beverages and sugary foods [15]. The photovoice training and discussion session recordings were professionally transcribed in the language in which they were conducted; bilingual team members then translated the Spanish language transcripts to English to facilitate analysis by non-Spanish speaking staff. Translated transcripts were then reviewed by another bilingual staff member and any identified discrepancies were adjudicated by the team of bilingual staff. To analyze the transcripts, we used a qualitative, team-based thematic analysis approach [12]. Team members consisted of a graduate student, a postdoctoral fellow in health psychology and two senior investigators experienced in qualitative methods. Two team members (B.H. and L.Q.) created a preliminary codebook, which included code names and definitions. Each transcript was then reviewed and coded independently using a deductive coding process by two different team members (C.S. and M.V.). In subsequent meetings, members of the research team (Y.F., B.H., L.Q., and M.V.) reviewed common codes and discussed discrepancies and merged, deleted, and added codes during this process. We used an Ecological Framework [10] depicting individual-, social-, physical-, and macro-level environmental influences on eating behaviors to organize our codes. We then grouped similar codes together to create themes. We used NVivo version 12 as our qualitative data management platform.

## Results

A total of 18 sessions (i.e., nine training and nine discussion) were recorded and transcribed, 12 were conducted in English (i.e., six training sessions and six discussion sessions) and six were conducted in Spanish (i.e., three training sessions and three discussion sessions). Participants attended only one training and one discussion session. Groups ranged in size, with between 2 to 4 participants. The average duration of the groups was 60 min. Demographic information about participants (*n* = 49) is shown in Table 1. Participants were predominantly women (83.7%) and identified as Hispanic (61.2%), followed by non-Hispanic Black (30.6%), non-Hispanic white (2.0%), and other (6.1%). In Boston, residents who identify as Hispanic ethnicity predominantly report being from the Dominican Republic or Puerto Rico.

**Table 1:** Characteristics of the study population (*n* = 49)

Characteristics		N / mean	% / SD
**Female Gender**		41	83.7%
**Language**			
English		21	43.1%
Spanish		16	33.3%
Both English & Spanish		12	23.0%
**Race/Ethnicity**			
Hispanic		30	61.2%
Black non-Hispanic		15	30.6%
White non-Hispanic		1	2.0%
Other		3	6.1%
**USA born**		19	38.8%
**Education**			
Less than High School		14	28.6%
High school graduate/GED		24	49.0%
Some college/technical school		9	18.4%
College graduate		2	4.0%
**Employment Status**			
Full Time		3	6.1%
Part time		12	24.5%
Unemployed		34	69.4%
**Self-reported diabetes**		6	12.2%
**Food insecurity**			
Worried about not having enough food		18	36.73%
Ate smaller meals because there wasn’t enough food		11	22.45%
Ate fewer meals in a day because of lack of food		8	16.33%
**Frequency of Sugary Drinks**			
Rarely or never		15	30.6%
Weekly		9	18.4%
Once a day		2	4.1%
Twice a day		9	18.4%
Three or more times a day		14	28.6%
**Frequency of Sugary Foods**			
Rarely or never		22	44.9%
Weekly		10	20.4%
Once a day		5	10.2%
Twice a day		5	10.2%
Three or more times a day		7	14.2%
**Knowledge about sugary beverages, score**^**a**^** (n(mean))**		49	1.4
**Self-efficacy on Sugary Drinks**^**b**^** (n(mean))**		48	3.5
**Self-efficacy on Sugary Foods**^**c**^** (n(mean))**		48	3.2
**Received SNAP**^**d**^		37	75.5%
**Self-rated dental health status**			
Excellent/Very Good/Good		24	49.1%
Fair/Poor		17	34.7%
Subject is edentulous		8	16.3%
**Self-rated health status**			
Excellent/Very Good/Good		26	53.1%
Fair/Poor	23		46.9%

^a^Scale represents 4 questions with multiple choice response options. For all questions, there was only one correct option. A correct response was assigned a 1 and an incorrect or ‘don’t know’ response was assigned a 0. Items are summed to create a knowledge score

^b,c^Each scale represents 5 questions with Likert scale response options, scored 1 to 5 and ranged from “Not sure at all” to “Extremely sure” for some self-efficacy questions and “Strongly disagree” to “Strongly agree” for other self-efficacy questions, depending on question content. Items are averaged to create a self-efficacy score

^d^*SNAP* Supplemental Nutrition Assistant Program

A higher proportion of participants reported consuming sugary beverages once per day or more (51.1%) compared to sugary foods (34.6%). Participants reported a moderate level of self-efficacy (mean score: 3.5 for limiting sugar-sweetened beverages and 3.2 for limiting sugary foods) on a scale from 1 (low self-efficacy) to 5 (high self-efficacy), while reported knowledge was low (average score was 1.4 correct out of 4 items).

To contextualize the themes in the wider dataset, in Table 2, we presented 34 codes according to the four levels of the multilevel Ecological Model (see also Fig. 1). The quantification of code occurrences serves to highlight which factors were most and least frequently mentioned across focus groups; in general, there were more individual-level factors compared to factors at the social, physical, or macro-environment levels. In Table 3, we presented the codes, along with selected participant photos, for three thematic categories used to summarize and describe participants’ perceptions of influences on their sugary food and beverage consumption.

**Table 2:** Summary of codes

Code	Definition	Number of code occurrences	Number of reference files (max = 18)
**Individual Factors**			
Health conditions	Relationship between diabetes and other health conditions like obesity (both personally and within the family) and food choices, maintaining health issues that they have, e.g., high blood pressure, family history of health conditions	42	10
Education or knowledge	Information about different topics	35	8
Value of fresh foods and home-cooked meals	Idea that meals prepared at home using fresh ingredients are superior in terms of healthfulness and other values	32	11
Psychological Factors	Anxiety, cognitive strategies, self-talk, emotional eating	19	8
Eating Behavior-Taste	Preference for healthier foods is influenced by how much you like them	19	7
Cost	Financial concerns, healthy foods perceived to be more expensive	16	4
Beliefs about food & nutrition	General beliefs about food and nutrition	14	6
Feeding strategies	Addresses kids not wanting healthy foods and ways to control your own intake and make food more appetizing	14	4
Self-Control	Self-control, willpower, commitment, self-empowerment	13	4
Eating Behavior-Cravings	Described need or craving for certain foods or drinks	12	6
Eating Behavior-General	Snacking, late night eating, meal skipping, habits, preferences	11	6
Substitution	Food swaps to increase healthfulness	10	4
Religion or Spirituality	Impact of religious beliefs on diet	9	4
Exercise as motivation	Doing physical activity influences future food choices	4	3
Boredom	Feeling bored and how this influences food choices	2	2
Follow Dr. Advice	Doctor’s advice to patients and their families about diet	2	2
**Social Environment**			
Education or knowledge	Information about different topics	35	8
Home environments	Situation at home and how it influences decisions, including screen time habits, dependency on others for cooking	32	10
Family and cultural traditions	Passing cooking and food choices down to future generations. Also includes how family members (not kids) influence food choices and availability	23	7
Roles of kids	They don’t eat/want the things you think they should. They hold you accountable to your goals. You may be more motivated to cook better for them than you would yourself	19	7
Social Support	Support from others for healthy behaviors	18	6
Work environments	Food at meetings, or donuts in the morning, and food offered by others in general is hard to turn down, work stress and anxiety,	12	5
Exercise as motivation	Doing physical activity influences future food choices	4	3
Social pressure	Pressure in social interactions to eat certain foods	1	1
**Physical Environment**			
Home environments	Situation at home and how it influences decisions, including screen time habits, dependency on others for cooking	32	10
Convenience	Factors driving food selection are based on perceived low amounts of time and how easy it would be to fulfill hunger for themselves or family	19	7
Cost	Financial concerns, healthy foods perceived to be more expensive	16	4
Work environments	Food at meetings, or donuts in the morning, and food offered by others in general is hard to turn down, work stress and anxiety	12	5
Wary about tap water	Concerns about drinking tap water	6	3
Neighborhood environments	Convenience stores (vs supermarkets); idea that SSBFs are pervasive, and access is everywhere	3	2
**Macro Environment**			
Cost	Financial concerns, healthy foods perceived to be more expensive	16	4
Family and cultural traditions	Passing cooking and food choices down to future generations. Also includes how family members (not kids) influence food choices and availability	23	7
Marketing	Advertising and packaging for kids, sales at the store that entice you to change choices, etc	26	9
Pandemic behavior	COVID-related influences on diet	14	9
Socio Political environment/food stamps	Policy level initiatives that impact choice and intake	5	4

**Table 3:**
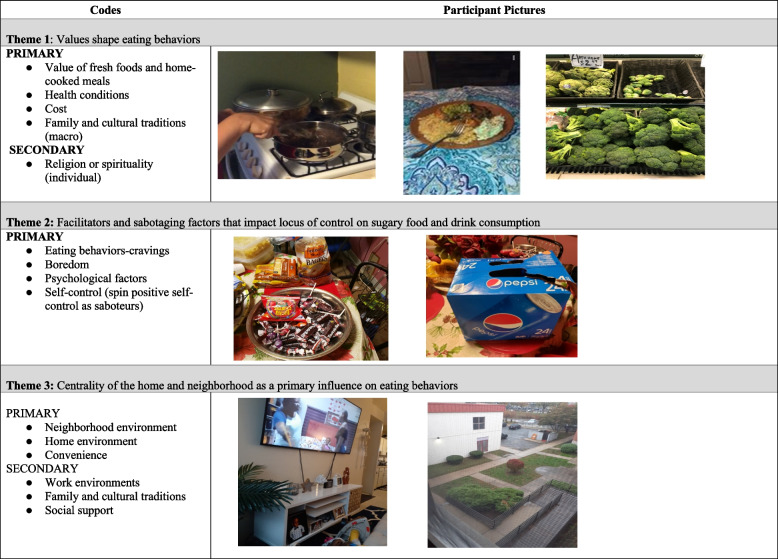
Qualitative theme description and participant photograph examples

### Theme 1: Values shape eating behaviors

On an individual level, participants valued fresh food and home cooked meals for a variety of reasons. Some residents reported fresh food and home cooked meals are beneficial to their overall health as well as the health of their kids. One participant took a photo of her mother cooking and stated, “*…I don’t make it a habit to eat out. We don’t eat – as it’s called, junk food. No, no, no. We don’t consume that.”* Another participant agreed and stated: “*I’m also the type of person who loves to cook at home; I don’t like to buy food on the street, much…It’s healthier, you use…healthy vegetables.*” A second idea participants shared is the influence of health conditions in shaping their eating behavior. Participants also highlighted how health conditions significantly shape their eating behaviors. Those with health conditions or family members with chronic health conditions expressed a strong preference for preparing meals at home using fresh ingredients. This practice was valued not only for its nutritional benefits but also for the sense of control it provided over their health conditions. By choosing to cook at home and use fresh foods, participants felt empowered to manage their health more effectively. One resident stated, “*as soon as you reach 30 you need to lower the levels of salt, sugar, oils, and everything you use to cook when you come from a family that is prone to getting diabetes.*” Participants mentioned that these benefits make using fresh food to cook meals at home worth it, despite the cost. For example, a participant took a photo of a grocery store aisle and when asked to share her reasoning behind the photo, *“Well,* [because] *I do love fruit. …most of the time when I do fruit shopping, I still get them even though they are expensive. I still get them* [participant placed emphasis on this phrase]*. I love banana and apple like every time we food shop I have to get them. So when I've seen them there I was like wow”.*

Participants also expressed societal and cultural social norms that exerted influence on their eating behaviors. For example, Hispanic participants expressed how their culture of origin influenced not only the type of food they cook, but also how they cook it. One participant stated, *“We are Dominicans; we move fast. So, we get something, we make rice and beans, uh, we put a lot of seasoning on it, you know what I mean? So, I have learned that it is better to cook with less seasoning and for everything to be natural, all natural.*” In another example, participants agreed current cultural norms affected their eating choices; for example, when viewing a photograph of a doughnut, one participant stated, “*Those are donuts that people usually eat in the morning for breakfast, mostly Americans. At my job, they always bring donuts, and it's sometimes very hard to say no.”* The influence of culture and social norms affecting eating behavior was common throughout the sessions.

### Theme 2. Facilitators and sabotaging factors that impact locus of control on sugary food and drink consumption

On the individual level, eating behavior (often in the form of cravings) was a common saboteur among participants trying to reduce their consumption of sugary food and sugary drinks. Participants felt eating behaviors they gained over the years are hard to break. For example, one participant stated the following: “*…I'm getting ready to be 50 years old in a couple of years. So, for me, I've been drinking water this way or, you know, I've been eating potatoes this way. I eat steak this way. Or – you know what I mean? So, for somebody to come and they say, "Oh, girl. You can't eat like that. You gotta cook it –" "No, honey. No. I've been eating like this since I was 5 years old."* Residents referenced many eating behaviors in the form of cravings for sugars. A participant took a photo of a box of soda, reflecting the idea expressed by many that sugar cravings like an addiction; for example, *“… people get addicted to drugs, some of us are addicted to soda.”* Participants often tried to combat cravings by relying on self-control, as expressed by one participant: *“It's hard to control it…But I try. My kids tell me not to eat this. Not to eat that. But,…it's hard when it's in front of you.”* Tension was expressed as a balance between a perceived lack of self-control, in which changing eating/drinking behaviors was hard to achieve; while for other participants, *“there has never* [been]* a barrier that you can't overcome”.*

### Theme 3: Centrality of the home and neighborhood as a primary influence on eating behaviors

The home as a place through which many positive and negative influences on participants' sugary food consumption are connected (termed centrality of the home) was a common theme. Several factors were consistent with influences at the social environment level. For example, participants agreed that social support from family and friends affected food consumption. One participant explained the vital role family played in their approach to cooking *“I'm not* [a] *big cooker in a way,…not a cook. I like I said, my sister, my daughter cook for me. So I'm blessed with that.”* Other participants also expressed family and cultural traditions in making food choices, *“And I do that, I share* [recipes] *with my family within my home, and with my family, my family circle I grew up with. So, I take it for my parents, for my siblings, and I recommend it to them. What I feel is beneficial to me, I recommend it – I push it forward. Because, I see it as a chain, I like for it to continue, you know, so that the…information gets further, and…the idea can be spread, the…knowledge. And I know that it’s something good; it’s something healthy.”*

As shown on Table 3 participants took photos’ of several other factors were consistent with influences at the neighborhood environment level, another commonly discussed topic was how proximity of the home to food stores affected choices. Participants indicated that due to busy schedules and family responsibilities, they will often rely on nearby food stores, often going to fast food or convenience stores to satisfy hunger. Often, these stores have fewer healthier options which are also not prominently displayed. One participant noted the following, “*for example Coca-Cola has…Dasani* [as] *the brand for the water but sometimes you don't even see the Dasani water, they only offer you in the machines the soda and I say why for that reason when I'm going out …from my house I used to take…water bottles from home because I know it's so hard and expensive to get…water bottle*[s] *on the street.”* There was agreement that busy schedules can sabotage their efforts in finding places that offer healthy food options. For example, a participant expressed her busy schedule influenced her eating choices, *“Oh, sometimes with work,…stress from work, lack of time,…lack of will power, lack of – Many things have an influence on you being fully on a diet or trying to keep on eating healthier. Sometimes here you do not…you do not have the time to do things to get better. I do think that that is a barrier and, well, you have to do your best to be able to,…eat healthy even you are very busy, because sometimes you are so busy that you forget about what you have to do.”*

## Discussion

Through the use of photovoice, residents of Boston public housing developments identified factors that influence their sugary food and beverage consumption. Based on an Ecological Framework, these factors range from the macro level, physical environment, social environment, to the individual level. Our findings consisted of three major themes: values shape eating behaviors, facilitators and sabotaging factors on sugary food and drink consumption, and the centrality of the home as a primary influence on eating behaviors. In prior research done by members of this team, we examined barriers and facilitators to diet, physical activity, and weight management among public housing residents.[12] Our current findings extend those findings by focusing more narrowly on sugary food and beverage consumption, an important defined modifiable dietary behavior, and by extending knowledge about cultural traditions through the inclusion of a large proportion of our sample as Spanish-speaking residents. Additionally, even though our discussion questions posed to participants focused on sugary foods and beverages, participants often returned to factors that influenced healthy eating and cooking more generally. This leads us to posit that in order to ultimately have an impact on sugary foods and beverage consumption, message design needs to focus on multi-level drivers of healthy eating, such as values, cultural traditions, and the home environment. In fact, a recent study looking at a sample of food consumers in the US found that the way in which consumers perceive the ‘healthfulness’ of a particular food product is evenly split between assessments of the particular food product as a healthy/unhealthy food, versus the food product as an indicator of the healthiness of one’s overall diet [19]. Thus, it is understandable that participants in our study may not have been thinking only about specific consumption patterns (e.g., sugary foods and beverages) independent of their overall approach to healthy eating.

These findings are consistent with prior research that examined influences on individual choices on healthy eating behaviors in general, but not necessarily sugary foods and beverages [6, 11–13]. Prior literature suggests factors such as family support influenced eating choices [12, 13]. In a study of rural women, family support was a positive influence in shaping their eating behaviors. The study further describes generational influences that mothers had on their children. Most of the studies focus on the influence of the physical environment, showing that location of residence influences food choices, often acting as barriers [12, 13]. One study [13] showed that location of food outlets created a space for members to eat, that did not translate into healthy eating.

Because individuals’ diet behaviors are directly and indirectly influenced by social and physical environments and psychosocial characteristics, collectively, these factors need to be appropriately accounted for in the development of culturally relevant intervention messaging. Our use of photovoice methods are uniquely suited to our goal of developing population-specific messaging around the reduction of sugary food and beverage consumption. Specifically, the photographs collected by residents, or representations of them, can assist in ensuring that messages accurately reflect images of people from target populations and the multi-level context in which target populations report that behaviors occur. This may enable more culturally relevant messages that are ultimately more effective in reducing sugary food and drink consumption. For example, Zhou and colleagues developed a series of messages targeting reduced sugary beverage consumption among Latino and African American youth that employed both surface and deep tailoring, and then examined perceptions of this tailoring among their target audience [20]. Our results reflect the body of research [13, 14, 21] indicating multiple environmental influences on healthy eating behaviors and is likely applicable to consumption of sugary foods and beverages.

Intervention research conducted in public housing settings supports the feasibility and preliminary efficacy of approaches that recognize the importance of environmental factors, including the social environment [22]. Intervention messages that arise from our findings should incorporate these themes: centrality of the home (and surrounding social and neighborhood areas) and take into account cultural influences (such as the value of fresh foods, family traditions, and health conditions) and individual-level sabotaging factors (like cravings and issues with self-control). Interventions that aim to address the physical and social environment (e.g., development-level implementation of cooking classes, mobile food bus, and community health worker-led walking groups) [23, 24] and social network interventions [25] may be ways to ‘move the needle’ on addressing these themes. Policy-level interventions (categorized as financial, advertising, healthy defaults, and availability such as healthy retail environments) that bring about equitable population wide changes can also incorporate these generated themes (for example advertising in retail environments that takes cultural influences into account) [26]. However, further research is needed to better understand through which delivery channel culturally relevant messages should be delivered. Options such as verbal/print dissemination through peer educators [22] or through text messaging [27] are promising as tested in feasibility studies in low-income populations; a study comparing the same message content across delivery channels may further elucidate which channel is most acceptable.

There may be a potential selection bias in that individuals agreeing to participate may be more likely to eat healthier foods and beverages. However, because about half of our sample reported consuming sugary drinks at least daily, we do not believe this was a substantial risk to the generalizability of our findings. Future studies could over-sample individuals who consume sugary drinks more frequently to mitigate potential selection bias. Due to the pandemic, participants may have been more restricted in their movement outdoors or in community spaces compared to their pre-pandemic lifestyles—this may have affected the places and scenarios where they took photographs. In an attempt to counter this effect, some participants presented photographs from online sources.

## Conlusions

As evidenced by the themes that emerged from the discussions of participant photos, choices around consumption of sugary foods and beverages appear to occur within a broader context that extend beyond individual preferences or behaviors, although those were also important. Specifically, the centrality of the home environment and the role of family and culture appeared to meaningfully shape values and influence behaviors around food selection and consumption. For this reason, it is likely important to acknowledge such influences when attempting to develop messaging or interventions that target food and beverage consumption behaviors.

## Data Availability

The datasets used and/or analyzed during the current study are available from the corresponding author on reasonable request.
